# Evaluation of a Novel Single-Tube Method for Extended Genotyping of Human Papillomavirus

**DOI:** 10.1128/JCM.01687-17

**Published:** 2018-02-22

**Authors:** R. Bhatia, I. Serrano, H. Wennington, C. Graham, H. Cubie, E. Boland, G. Fu, K. Cuschieri

**Affiliations:** a HPV Research Group, University of Edinburgh, Edinburgh, United Kingdom; b Edinburgh Clinical Research Facility, University of Edinburgh, Edinburgh, United Kingdom; c Global Health Academy, University of Edinburgh, Edinburgh, United Kingdom; d Genefirst, Oxford, United Kingdom; e Scottish HPV Reference laboratory, Royal Infirmary of Edinburgh, NHS Lothian, Edinburgh, United Kingdom; Memorial Sloan Kettering Cancer Center

**Keywords:** HR-HPV, cervical screening, genotyping, multiplex probe amplification

## Abstract

The use of high-risk human papillomavirus (HPV) testing for surveillance and clinical applications is increasing globally, and it is important that tests are evaluated to ensure they are fit for this purpose. In this study, the performance of a new HPV genotyping test, the Papilloplex high-risk HPV (HR-HPV) test, was compared to two well-established genotyping tests. Preliminary clinical performance was also ascertained for the detection of CIN2+ in a disease-enriched retrospective cohort. A panel of 500 cervical liquid-based cytology samples with known clinical outcomes were tested by the Papilloplex HR-HPV test. Analytical concordance was compared to two assays: a Linear Array (LA) HPV genotyping test and an Optiplex HPV genotyping test. The initial clinical performance for the detection for CIN2+ samples was performed and compared to that of two clinically validated HPV tests: a RealTime High-Risk HPV test (RealTime) and a Hybrid Capture 2 HPV test (HC2). High agreement for HR-HPV was observed between the Papilloplex and LA and Optiplex HPV tests (97 and 95%, respectively), with kappa values for HPV16 and HPV18 being 0.90 and 0.81 compared to the LA and 0.70 and 0.82 compared to the Optiplex test. The sensitivity, specificity, positive predictive value, and negative predictive value of the Papilloplex test for the detection of CIN2+ were 92, 54, 33, and 96%, respectively, and very similar to the values observed with RealTime and HC2. The Papilloplex HR-HPV test demonstrated a analytical performance similar to those of the two HPV genotyping tests at the HR-HPV level and the type-specific level. The preliminary data on clinical performance look encouraging, although further longitudinal studies within screening populations are required to confirm these findings.

## INTRODUCTION

The use of high-risk human papillomavirus (HR-HPV) testing for the identification of women at risk of developing cervical cancer and for the management of women who have received treatment is increasing globally (1). In addition, type-specific HPV detection methods are valuable both for epidemiological studies and as a triage for primary HR-HPV infection (2). There are now a wide variety of commercially available HPV tests (3) that vary in terms of detection chemistry, complexity, type range, throughput, and required equipment. Although a component has been clinically validated for use in primary HPV screening through assessment according to internationally accepted criteria or used extensively in longitudinal research and surveillance endeavors, peer-reviewed evidence on the analytical and/or clinical performance of several tests is lacking.

The Papilloplex HR-HPV test (Genefirst, UK) is a commercially available HPV genotyping test that performs quantitative multiplex detection of 14 HR-HPV types, together with an endogenous human control target, in a single tube (4). Based on multiplex probe amplification (MPA) technology, the assay utilizes differing melting-curve profiles to allow the differentiation of up to six targets per fluorescence channel within a real-time assay (4). The test is compatible with real-time PCR equipment commonly used in clinical and research laboratories and so does not require a specific locked-down platforms.

We present here results from an evaluation of the Papilloplex HR-HPV assay in which its performance is compared to two qualitative, broad-spectrum, extended genotyping assays: the Linear Array HPV genotyping test (LA; Roche Molecular Systems, Inc., Alameda, CA) and the Optiplex HPV genotyping kit (formerly the Multiplex HPV genotyping kit; DiaMex, Heidelberg, Germany). Preliminary insight into clinical performance of the assay is also presented through its ability to detect CIN2+ in a disease-enriched sample compared to two well-established clinically validated HPV assays: the Hybrid Capture 2 HPV DNA test (HC2; Qiagen, Inc., Gaithersburg, MD) and the RealTime High-Risk HPV test (RealTime; Abbott Molecular, Des Plaines, IL).

## MATERIALS AND METHODS

### Samples and approvals.

A total of 500 liquid-based cytology samples (LBCs) were obtained from the Scottish HPV Archive (www.shine.mvm.ed.ac.uk/archive.shtml), which is a biobank designed to support HPV Research. The East of Scotland Research Ethics Service has given generic approval to the Scottish HPV Archive as a research tissue bank (REC Ref 11/AL/0174) for HPV-related research on archived samples. The Scottish HPV Archive is also registered with National Research Scotland (NRS) Lothian Bioresource. Samples were made available for the present project through application to the archive steering committee (HPV Archive Application Ref 0016).

The samples used for the study included 473 samples collected from women attending their first routine smear at the age of 20 in Scotland, supplemented by 27 samples from women attending colposcopy clinics due to abnormal cytology (in order to enrich for CIN2+). Routine cytology classification was according to British Society for Clinical Cytology criteria (5). Cytology results were classed as negative (for any abnormality), low grade (borderline squamous changes, koilocytosis, and low grade dyskaryosis), and high grade (which includes moderate and severe dyskaryosis). Subsequent cytology and histology results were obtained through data linkage via Information Services Division, Scotland, and samples were classified as 2× cytology negative (with two subsequent negative cytology results at least 1 year apart), ≤CIN1 or CIN2+ (Table 1). Samples had originally been collected between 2010 and 2012 and stored in the archive at −80°C.

### HPV DNA testing.

Samples were retrieved and aliquots prepared for HPV testing with the Papilloplex HR-HPV test, HC2, Optiplex HPV genotyping test, LA, and RealTime HR-HPV test. The Papilloplex test was performed in Genefirst laboratories (Oxford, United Kingdom). All other tests were performed at the Scottish HPV Reference Laboratory and HPV Research Group (Edinburgh). All tests were performed according to the manufacturer's instructions, although a brief description of assay characteristics is provided in Table 2 and a detailed description of the Papilloplex HR-HPV test is provided in the next section. The Optiplex genotyping test has been used for longitudinal immunization surveillance in Scotland (6–8) and has been adjudicated as proficient for detection of HPV types 6, 11, 16, 18, 31, 33, 35, 39, 45, 51, 52, 56, 58, 59, 66, and 68 according to the last three consecutive World Health Organization laboratory network (WHO LabNet) HPV DNA proficiency schemes (when testing was performed in Edinburgh). LA is also associated with good performance on WHO LabNet proficiency panels as outlined in Eklund et al. (9), where it was the most frequently applied assay to the scheme.

### Papilloplex HPV test.

The Papilloplex HR-HPV test was performed on DNA extracted using two different methods. Half the samples were extracted using a QiaAmp DNA minikit (Qiagen, Germany) and half using the automated Nuclisens EasyMag system (bioMérieux, France). The method of extraction was randomly allocated to samples.

A total of 2 μl of DNA was added to the PCR amplification reaction mix (18 μl) containing buffer (deoxynucleoside triphosphates and Mg^2+^), master mix (*Taq* polymerase, UNG enzyme, and dUTP) and working mix (primers and probes) to obtain a final volume of 20 μl per PCR. The PCR was performed on ABI 7500 Fast real-time PCR systems (Applied Biosystems, Warrington, United Kingdom). The thermal profile was set as follows: amplification stage 1, 50°C for 2 min, followed by 95°C for 3 min; amplification stage 2, 9 cycles of 95°C for 6 s, followed by 66°C for 45 s; and amplification stage 3, 42 cycles of 95°C for 3 s, followed by 60°C for 33 s, and 63°C for 15 s. Fluorescence measurements in the ROX, FAM, HEX (JOE), and CY5 channels were recorded during step 2 of amplification stage 3 (60°C for 33 s). A preset dissociation stage (stage 4) was included following the final PCR cycle of amplification (stage 3). The postamplification melting profile protocol comprised 95°C for 15 s, 25°C for 1 min, 75°C for 15 s, and 60°C for 15 s. The fluorescence emission data were continually collected during the temperature increase. The negative derivative of the emission reading, with respect to temperature, was plotted against the temperature to form melting curves (per fluorescent channel) generated during the dissociation stage of the reaction (from 25 to 75°C).

For consistency between experiments, the following threshold values for *C_T_* determination were set (ROX, 100,000; FAM, 100,000; HEX, 25,000; and CY5, 50,000). For each sample, the internal control (CY5 detection channel) and all 14 HR-HPV types, corresponding to the ROX (HR-HPV types 33, 35, 45, 51, 56, and 66), FAM (HR-HPV types 16, 18, 31, 52, and 59), and HEX channel (HR-HPV types 39, 58, and 68) were simultaneously evaluated. Samples were considered positive for HR-HPV DNA types if a *C_T_* value was <38 for cellular DNA and <36 in any of the ROX, FAM, and HEX fluorescent channels. A sample was considered invalid if the *C_T_* value of cellular DNA was >38. The change in the characteristic melting profile(s) in the sample was compared to the negative-control reference melting profile to identify the genotypes present. Samples were tested in batches of 96 samples (including controls) per reaction.

### Analysis. (i) HR-HPV concordance of the Papilloplex with comparator tests.

Type-specific positivity for each HR-HPV type included in Papilloplex was compared to the Optiplex and LA. Concordance, proportional agreement with accompanying 95% confidence intervals (CI) have been presented, along with kappa statistics and the McNemar test. The Papilloplex was also compared to the above-described tests at the level of HR-HPV positivity (for the types covered by Papilloplex only).

### (ii) Assessment of preliminary clinical performance.

Clinical performance of the Papilloplex test was measured as sensitivity, specificity, positive predictive value, and negative predictive value for the detection of cervical CIN2+ with 95% CIs around the percentages. The clinical performance of the HC2 and RealTime HPV test was also performed and presented alongside the Papilloplex results. Disease cases were defined as CIN2+ (*n* = 87), whereas no disease was defined as histologically confirmed CIN1 or less or a sample being associated with two consecutive negative cytology results at least 1 year apart (*n* = 349). Pathology data were incomplete to allow this categorization for 64/500 samples so clinical performance assessment was performed on 436 samples.

## RESULTS

### Overall HR-HPV positivity in the cohort.

The study cohort consisted of 500 Thinprep LBC samples with known cytology and histology results (Table 1). The sample cohort of 500 was split into two extraction methods (250 extracted using a manual QiaAmp DNA minikit and 250 using an automated Nuclisens EasyMag system). The concordance of Papilloplex at overall HR-HPV level and type-specific level with LA and Optiplex showed no significant differences based on extraction chemistry (data not shown). The whole study cohort was therefore used for further analysis. Overall HR-HPV positivity for the genotyping tests and those for the clinically validated tests were similar: 58.4% for Papilloplex, 57.2% for LA, 56.4% for Optiplex, 56.2% for RealTime, and 58.6% for HC2 (Table 2).

**TABLE 1:** Cervical pathology associated with study population^*a*^

Pathology	No. (%) of samples
Underlying cytology	
Negative	266 (53.2)
Low-grade dyskaryosis	156 (31.2)
High-grade dyskaryosis	66 (13.2)
Unknown	12 (2.4)
Total	500
Underlying histology	
No histology performed (2⇆ negative cytology)	263 (52.6)
≤CIN1	86 (17.2)
CIN2+	87 (17.4)
Histology information incomplete	64 (12.8)

a Note that clinical performance assessment was performed on 436 samples. Samples with incomplete histology were not included in this analysis.

**TABLE 2:** Description of assays used in the study with the detection technology, types covered, and prevalence of HPV in the study population

Test	Detection technology	HPV types identified by the test		No. (%) of samples	
		High risk	Low risk	High risk positive	High risk + low risk positive
Papilloplex HR- HPV test	Real-time PCR with individual genotyping	16, 18, 31, 33, 35, 39, 45, 51, 52, 56, 58, 59, 66, 68		292 (58.4)	
RealTime HR-HPV test	Real-time PCR with partial genotyping	16, 18, 31, 33, 35, 39, 45, 51, 52, 56, 58, 59, 66, 68		281 (56.2)	
Hybrid Capture 2	Target amplification followed by Sandwich capture assay	16, 18, 31, 33, 35, 39, 45, 51, 52, 56, 58, 59, 68 [not 66]		293 (58.6)	
Linear Array HPV genotyping test	Target amplification followed by hybridization	16, 18, 31, 33, 35, 39, 45, 51, 52, 56, 58, 59, 66, 68	6, 11, 26, 40, 42, 53, 54, 55, 61, 62, 64, 67, 69, 70, 71, 72, 73, 81, 82, 83, 84, IS*39*, CP6108	286 (57.2)	340 (68.0)
Optiplex HPV genotyping test	Target amplification followed by luminex detection	16, 18, 31, 33, 35, 39, 45, 51, 52, 56, 58, 59, 66, 68	6, 11, 26, 42, 43, 44, 53, 70, 73, 82	282 (56.4)	321 (64.2)

### Agreement between assays.

The agreement of the overall HR-HPV positivity between Papilloplex and the two extended genotyping tests is shown in Table 3. High proportional agreement of 97% (95% CI = 95 to 98) was observed between the Papilloplex and the LA. Similarly, high proportional agreement of 95% (95% CI = 92 to 97) was observed between the Papilloplex and the Optiplex.

**TABLE 3:** Overall agreement between Papilloplex HR-HPV test and comparator tests^*a*^

Test	Status	Negative (no.)	Positive (no.)	% proportional agreement (CI)	Kappa	McNemar test (*P*)
Linear Array (LA) HPV Genotyping test						
Papilloplex HR- HPV test	Negative	203	5	97 (95–98)	0.934	0.210
	Positive	11	281			
Optiplex HPV genotyping test						
Papilloplex HR- HPV test	Negative	200	8	95 (92–97)	0.894	0.076
	Positive	18	274			

a Concordance between the samples is indicated, and the proportional agreement with the 95% CI (in parentheses), kappa, and McNemar test *P* values are listed.

Type-specific concordance between the Papilloplex and the two genotyping assays for HR-HPV types 16, 18, 31, 33, 35, 39, 45, 51, 52, 56, 58, 59, 66, and 68 is shown in Table 4. Two-by-two tables for each type detected by Papilloplex (versus comparator test) are also presented in the supplemental material (see Table S1). When comparing the Papilloplex to the Optiplex test, there was at least “substantial” agreement (defined according to a kappa value of 0.61 to 0.80) for all types except HPV68 (0.548). The equivalent comparison of Papilloplex to LA showed at least substantial agreement (defined according to a kappa value of 0.61 to 0.80) for all types except HPV68 (0.573) and HPV59, which at a kappa value of 0.614 was at the lower end of substantial agreement. Papilloplex detected fewer samples as positive for HPV16 (*n* = 98) compared to both LA (*n* = 108) and Optiplex (*n* = 146). Similarly for HPV59, Papilloplex detected fewer samples as positive (*n* = 20) compared to LA (*n* = 73) and Optiplex (*n* = 28), which is reflected in the aforementioned kappa value. Conversely, Papilloplex detected a higher number of HPV31 (*n* = 64) infections compared to the LA (*n* = 54) and Optiplex (*n* = 40) tests, and a higher number of HPV33 (*n* = 44) infections versus Optiplex (*n* = 36). Papilloplex also detected a higher number of HPV56 (*n* = 32) infections compared to LA (*n* = 22), but this was lower than those detected by Optiplex (*n* = 43) (see Table S1 in the supplemental material).

**TABLE 4:** Type-specific agreement of Papilloplex with Optiplex and Linear Array HPV tests^*a*^

HPV type and parameter	Optiplex HPV test	Linear Array HPV test
HPV16		
Proportional agreement	89 (86–91)	97 (95–98)
Kappa	0.7	0.902
McNemar test (*P*)	<0.001	0.021
HPV18		
Proportional agreement	97 (95–98)	97 (95–98)
Kappa	0.822	0.809
McNemar test (*P*)	0.286	0.077
HPV31		
Proportional agreement	95 (93–97)	97 (95–98)
Kappa	0.744	0.846
McNemar test (*P*)	<0.001	0.021
HPV33		
Proportional agreement	98 (97–99)	99 (97–99)
Kappa	0.966	0.91
McNemar test (*P*)	0.008	0.453
HPV35		
Proportional agreement	99 (98–100)	100 (99–100)
Kappa	0.774	0.907
McNemar test (*P*)	0.125	1
HPV39		
Proportional agreement	97 (96–99)	98 (96–99)
Kappa	0.851	0.937
McNemar test (*P*)	0.774	0.388
HPV45		
Proportional agreement	99 (96–99)	99 (98–100)
Kappa	0.867	0.924
McNemar test (*P*)	1	1
HPV51		
Proportional agreement	98 (96–99)	98 (97–99)
Kappa	0.879	0.914
McNemar test (*P*)	1	0.727
HPV52		
Proportional agreement	96 (94–97)	–^*b*^
Kappa	0.811	–
McNemar test (*P*)	0.664	–
HPV56		
Proportional agreement	97 (95–98)	98 (96–99)
Kappa	0.784	0.805
McNemar test (*P*)	0.007	0.002
HPV58		
Proportional agreement	98 (96–99)	98 (97–99)
Kappa	0.811	0.886
McNemar test (*P*)	0.146	0.727
HPV59		
Proportional agreement	98 (96–99)	95 (93–97)
Kappa	0.738	0.614
McNemar test (*P*)	0.039	<0.001
HPV66		
Proportional agreement	99 (97–100)	99 (97–99)
Kappa	0.915	0.908
McNemar test (*P*)	1	0.016
HPV68		
Proportional agreement	98 (97–99)	98 (96–99)
Kappa	0.548	0.573
McNemar test (*P*)	0.07	1

a The proportional agreement (%) with 95% CI (in parentheses), kappa, and McNemar test *P* values are indicated.

b –, Linear Array was unable to identify HPV-52 status in samples also positive for HPV33, HPV35, and/or HPV58. The results for HPV-52 are therefore not presented.

### Clinical performance for detection of cervical intraepithelial neoplasia 2 or worse (CIN2+).

Of the 500 samples in the panel, 87 were associated with CIN2+. The sensitivity, specificity, positive predictive value (PPV), and negative predictive value (NPV) results of the Papilloplex test for the detection of CIN2+ are summarized in Table 5, with values of 92, 54, 33, and 96, respectively. These values were similar to the clinical performance of the HC2 and RealTime assays.

**TABLE 5:** Clinical performance of HPV tests for detection of CIN2+^*a*^

Parameter	% (CI)		
	Papilloplex HR- HPV test	Hybrid Capture 2 (HC2)	RealTime HR-HPV test
Sensitivity	92 (84–97)	91 (83–96)	91 (83–96)
Specificity	54 (48–59)	54 (48–59)	56 (50–61)
PPV	33 (27–39)	33 (27–39)	34 (28–40)
NPV	96 (93–99)	96 (92–98)	96 (92–98)

a The sensitivity, specificity, positive predictive value (PPV), and negative predictive value (NPV), along with the 95% CI (in parentheses), are indicated.

## DISCUSSION

The Papilloplex HR-HPV test is a single-tube test for the quantitative multiplex detection of 14 HR-HPV types, together with an endogenous human control target. This study provides the first analytical assessment of the Papilloplex test compared to two commercially available HPV tests that offer extended genotyping capability: LA and Optiplex. Further, to gain insight into the potential clinical performance of the assay, a preliminary evaluation was undertaken to determine its ability to detect CIN2+ in a disease-enriched population.

Papilloplex showed high concordance to the Optiplex and LA tests at the level of overall HR-HPV positivity with a proportional agreement of 95 to 97% and a kappa of 0.90 to 0.93. Type-specific proportional agreement for all 14 HR-HPV types covered by Papilloplex was generally high, although there were some type-specific differences. Papilloplex showed moderate concordance to LA and Optiplex for HPV types 16 and 59, detecting less infection, and clearly, HPV16 is an important type for both epidemiological and clinical applications. On the other hand, Papilloplex detected more HPV31 infections compared to both comparator genotyping tests. Type-specific differences between genotyping tests have been reported previously (10), and such differences are perhaps inevitable given the range of chemistries available. Nevertheless, these data reinforce the notion that for longitudinal surveillance exercises (in which monitoring prevalence and trends of HPV types is important), consistent use of the same test is important to avoid real changes being confounded by test chemistry. Furthermore, it is notable that the clinical performance of the Papilloplex assay was similar to that of two well-established clinically validated tests, indicating that type-specific differences (including for HPV16) may not have significant implications for the detection of disease.

This said, we accept that the clinical evaluation performed in this study was preliminary and that the sample used was enriched in nature and did not represent women from a cross section of the screening population. Consequently, the clinical performance observed in this study is not representative of performance in a screening population. Nevertheless, determining initial sensitivity (the key measure of performance for screening applications) of a novel HPV test for CIN2+ using a sample with high disease prevalence has precedent (9, 11) and arguably showing performance relative to that of an assay in which clinical efficacy has been demonstrated also has value, even at an early stage. Furthermore, future clinical validation of the test which builds on the present work but involves a longitudinal screening population and assessment according to internationally recognized validation criteria is planned (12, 13).

The variety of HPV tests available with their different scopes and capabilities provides users with options to choose the most appropriate test for a particular context and population. Papilloplex HPV is a single-tube assay that identifies 14 HR-HPV types. The ability to perform individual genotyping within a single closed-tube format reduces time and the risk of contamination associated with more “open” genotyping systems. The assay is amenable to several DNA extraction chemistries, requires a small amount of input DNA, and can be performed with existing real-time 96-well PCR platforms that are available in routine research and clinical laboratories. In terms of analytical performances, we have shown that this assay compares favorably to existing, more-established extended genotyping assays. Although initial data on clinical performance are encouraging, further longitudinal assessments will determine its potential use for screening and disease management.

## Supplementary Material

Supplemental material
