# Supplementary material for: Evaluation of a Novel Single-Tube Method for Extended Genotyping of Human Papillomavirus
Source: J Clin Microbiol. 2018 Feb 22;56(3):e01687-17. doi: 10.1128/JCM.01687-17 (PMC5824038; doi:10.1128/JCM.01687-17)
Supplement: Supplemental material [file supp_56_3_e01687-17__index.html]

Supplemental material 

# Evaluation of a Novel Single-Tube Method for Extended Genotyping of Human Papillomavirus

## Supplemental material

- Supplemental file 1 -

  Table S1 (Type-specific agreement of Papilloplex results with those of Linear Array and Optiplex HPV tests)

  XLSX, 17K
